# Comparison of ChatGPT and Internet Research for Clinical Research and Decision-Making in Occupational Medicine: Randomized Controlled Trial

**DOI:** 10.2196/63857

**Published:** 2025-05-20

**Authors:** Felix A Weuthen, Nelly Otte, Hanif Krabbe, Thomas Kraus, Julia Krabbe

**Affiliations:** 1 Institute of Occupational, Social and Environmental Medicine, Medical Faculty, Rheinisch-Westfälische Technische Hochschule Aachen University, Aachen, Germany; 2 Department of Vascular Surgery, St. Josef Hospital Bochum, Katholisches Klinikum Bochum, Medical Faculty, Ruhr University Bochum, Bochum, Germany; 3 Institute for Prevention and Occupational Medicine of the German Social Accident Insurance, Medical Faculty, Ruhr University Bochum, Bürkle de la Camp Platz 1, Bochum, 44789, Germany, 49 3013001 ext 4110

**Keywords:** occupational diseases, artificial intelligence, internet research, medicine, occupational medicine, ChatGPT, AI, decision-making, algorithms, algorithm, large language models, LLMs, physicians, medical students, occupational lung diseases, occupational lung disease

## Abstract

**Background:**

Artificial intelligence is becoming a part of daily life and the medical field. Generative artificial intelligence models, such as GPT-4 and ChatGPT, are experiencing a surge in popularity due to their enhanced performance and reliability. However, the application of these models in specialized domains, such as occupational medicine, remains largely unexplored.

**Objective:**

This study aims to assess the potential suitability of a generative large language model, such as ChatGPT, as a support tool for medical research and even clinical decisions in occupational medicine in Germany.

**Methods:**

In this randomized controlled study, the usability of ChatGPT for medical research and clinical decision-making was investigated using a web application developed for this purpose. Eligibility criteria were being a physician or medical student. Participants (N=56) were asked to work on 3 cases of occupational lung diseases and answer case-related questions. They were allocated via coin weighted for proportions of physicians in each group into 2 groups. One group researched the cases using an integrated chat application similar to ChatGPT based on the latest GPT-4-Turbo model, while the other used their usual research methods, such as Google, Amboss, or DocCheck. The primary outcome was case performance based on correct answers, while secondary outcomes included changes in specific question accuracy and self-assessed occupational medicine expertise before and after case processing. Group assignment was not traditionally blinded, as the chat window indicated membership; participants only knew the study examined web-based research, not group specifics.

**Results:**

Participants of the ChatGPT group (n=27) showed better performance in specific research, for example, for potentially hazardous substances or activities (eg, case 1: ChatGPT group 2.5 hazardous substances that cause pleural changes versus 1.8 in a group with own research; *P*=.01; Cohen *r*=–0.38), and led to an increase in self-assessment with regard to specialist knowledge (from 3.9 to 3.4 in the ChatGPT group vs from 3.5 to 3.4 in the own research group; German school grades between 1=very good and 6=unsatisfactory; *P*=.047). However, clinical decisions, for example, whether an occupational disease report should be filed, were more often made correctly as a result of the participant’s own research (n=29; eg, case 1: Should an occupational disease report be filed? Yes for 7 participants in the ChatGPT group vs 14 in their own research group; *P*=.007; odds ratio 6.00, 95% CI 1.54‐23.36).

**Conclusions:**

ChatGPT can be a useful tool for targeted medical research, even for rather specific questions in occupational medicine regarding occupational diseases. However, clinical decisions should currently only be supported and not made by the large language model. Future systems should be critically assessed, even if the initial results are promising.

## Introduction

The application of artificial intelligence in the field of medicine has a long history, dating back to the mid-20th century. Initially used in research, its use in clinical medicine emerged in the 1970s. The MYCIN computer expert system was used at Stanford University for the purpose of diagnosing and treating infectious diseases with antibiotics [1]. Even though the diagnoses produced by the system at that time exhibited remarkably high success rates, it was not accepted at that time. The recent rapid innovation of large language models (LLMs) has led to the emergence of ChatGPT, which is the first LLM to provide the data basis and performance to support or carry out medical research and clinical decisions. Nevertheless, the clinical application is currently viewed with a degree of skepticism, as ChatGPT, especially in the earlier versions 2 and 3, demonstrated a marked tendency to “confabulate,” to fabricate statements and even references [2]. This phenomenon is frequently referred to as “hallucinating” in the literature [3]. Following the upgrade to ChatGPT 4, which includes a Bing internet connection, the tendency to hallucinate has been reported to have decreased significantly, allowing well-founded statements to be made. However, it is essential to subject LLM information to rigorous scrutiny and verification for accuracy.

ChatGPT has been used in patient care for some time, for example, for the creation of informational pamphlets [4], the evaluation of educational videos [5], and also in radiological [6] or dermatological [7] diagnostics. Nevertheless, there are currently significant discrepancies between the assessments of the relevant experts, and therefore, the use of this technology without proper oversight is not recommended [4,5,7]. Occupational medicine is a small specialty at the interface between work and medicine. One main field of activity is the prevention or early detection of occupational diseases. In Germany, an occupational disease can only be recognized officially if there is a disease ”that insured employees suffer as a consequence of the occupational activities they perform in the course of their jobs and that are listed in the Ordinance on Occupational Diseases in Germany [8]. Almost 80% of annual deaths are caused by occupational diseases of the lungs in Germany [9]. However, particularly nonoccupational physicians are frequently uncertain as to whether the clinical presentation of patients and their occupational history justify the reporting of an occupational disease. Furthermore, the results of an internet search are often inconclusive, particularly with regard to German occupational disease law.

This project originated from the clinical experience and routine of an occupational medicine institute at a university hospital, as well as interactions with colleagues during consultations. An application that facilitates targeted research or even indicates whether there is a justified suspicion of an occupational disease could have a positive impact on the daily work of doctors and on the probable high number of unreported occupational illnesses that are not reported.

The objective of this study was to assess the potential suitability of generative LLM, such as ChatGPT, as a support tool for medical research and even clinical decisions in occupational medicine in Germany. In particular, the first insights into the potential for such technology to provide assistance with questions pertaining to occupational disease law and the practice of daily medical care should be provided. Physicians and medical students were invited to work on 3 occupational medicine cases regarding occupational lung diseases within a web-based application. One randomly selected group was prompted to use an integrated chat application with input in ChatGPT, whereas the other was instructed to use their customary research instruments, including web-based search engines such as Google and, in Germany frequently used medical information websites such as Amboss [10] or DocCheck [11]. The responses provided were subjected to quantitative assessment based on the number of correct answers.

## Methods

### Participants

In this randomized controlled study, medical students and doctors were recruited via announcements on notice boards and personal contact. Flyers with information about the study and a QR code to the study website were shared in web-based and analog notice boards, as well as student messenger groups at the university hospital. Flyers were also distributed in other hospitals and rescue helicopter stations via contact persons who received the flyers and hung them on the notice boards. The web-based study was conducted in German via a web-based application. The inclusion criterion was the indication of current medical studies and semester or practice as a physician and specialty. An exclusion criterion was the use of ChatGPT in the group, which should use their own research tools. To avoid unconscious influence toward the use of ChatGPT, the participants were not informed of this at the start of the study. No specific sample size calculation was carried out beforehand on the assumption that recruitment per se would be rather difficult due to the effort involved. A minimum of 50 participants was set as a minimum number in a 3-month interval.

With a sum of 3 weeks of lectures, occupational medicine is a very small part of the study program at medical universities. Physicians and medical students both were expected to have a similar level of knowledge in relation to occupational medicine, since occupational health aspects play only a very small role, if any, in specialist medical training. Thus, both groups were included in the study.

**Figure 1.**
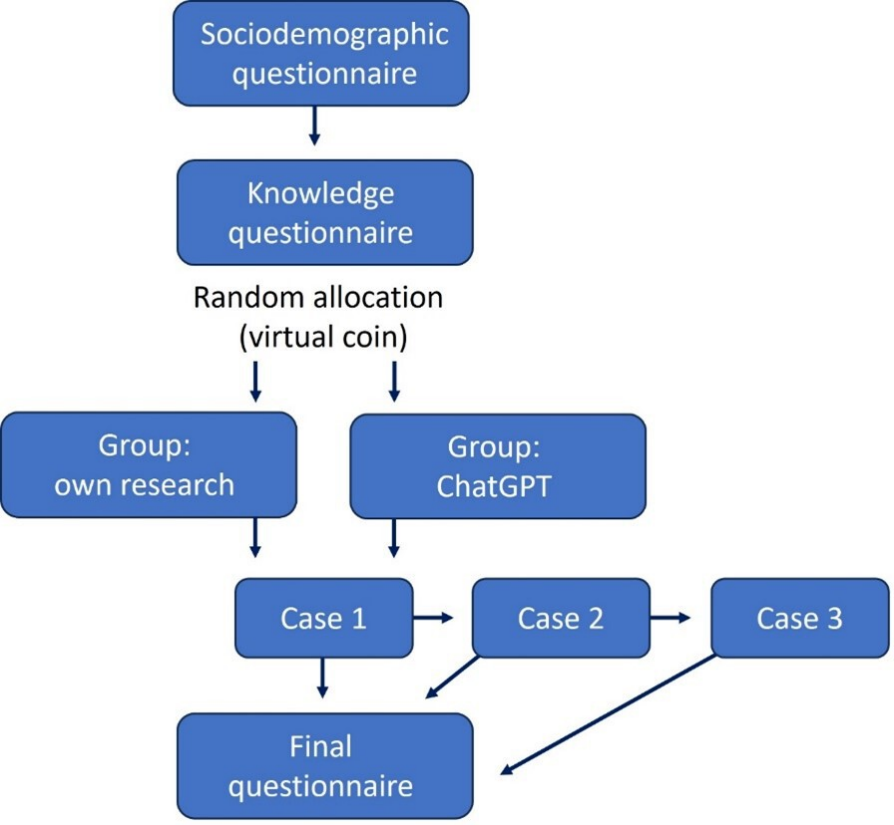
Study procedure. After the sociodemographic and knowledge questionnaire, participants were assigned to one of the 2 groups. After processing of case 1, participants could decide if they work on case 2 or proceed to the final questionnaire. The same choice was given after case 2.

The study process is depicted in Figure 1. As the first step, demographic data and a self-assessment of occupational medical knowledge were requested according to German school grades between 1 and 6, with 1=very good being the best grade and 6=unsatisfactory being the worst. The respondents were then asked 6 questions on occupational diseases, which they were asked to answer from memory without any research.

They were then randomized into one of two groups: (1) research with the integrated chat window, which enabled a query with ChatGPT, or (2) research with the research tools familiar to the person. The person was free to choose which tools were used; they were only asked to name these tools after the case studies. A digital weighted coin was flipped for the group assignment. If there was an unequal distribution of doctors and students, the next group assignment with the next coin toss was more likely to be allocated to the other group. Afterward, the participants were immediately referred to case processing.

The group assignment was not blinded in the traditional way, since group membership was indicated by the presence or absence of the chat window. However, the participants did not learn the exact group characteristics in advance only that web-based research was to be examined.

### Questions and Cases

The study was conducted between March 1, 2024, and May 31, 2024, as planned for a 3-month period. The English translation of the survey conducted in German can be found in Multimedia Appendix 1. Three cases were offered, each with 6 questions. All 3 cases were based on real patients in occupational medicine practice and only slightly altered for the study. Cases were then examined for accuracy by 2 occupational health specialists who specialize in occupational lung diseases. The correct answers were determined in advance. A pilot test with the setup was tested by 3 pilot testers, a medical student, a nonoccupational physician, and an occupational health specialist. According to their comments, slight adjustments were made, for example, copying the questions directly into the chat window was disabled.

Case 1 is based on the case of an outdoor worker who was treated for cholangiocellular carcinoma. As an incidental finding, asbestos-associated changes in the pleura were found in the computed tomography of the thorax. An occupational disease report was made with a justified suspicion of an occupational disease according to the Occupational Diseases Ordinance in Germany. Case 2 is based on the case of a young woman who worked in galvanization and developed a sensitization and allergy to a metal sulfate. Case 3 was based on a former dental technician with a recognized occupational disease (berylliosis).

**Figure 2.**
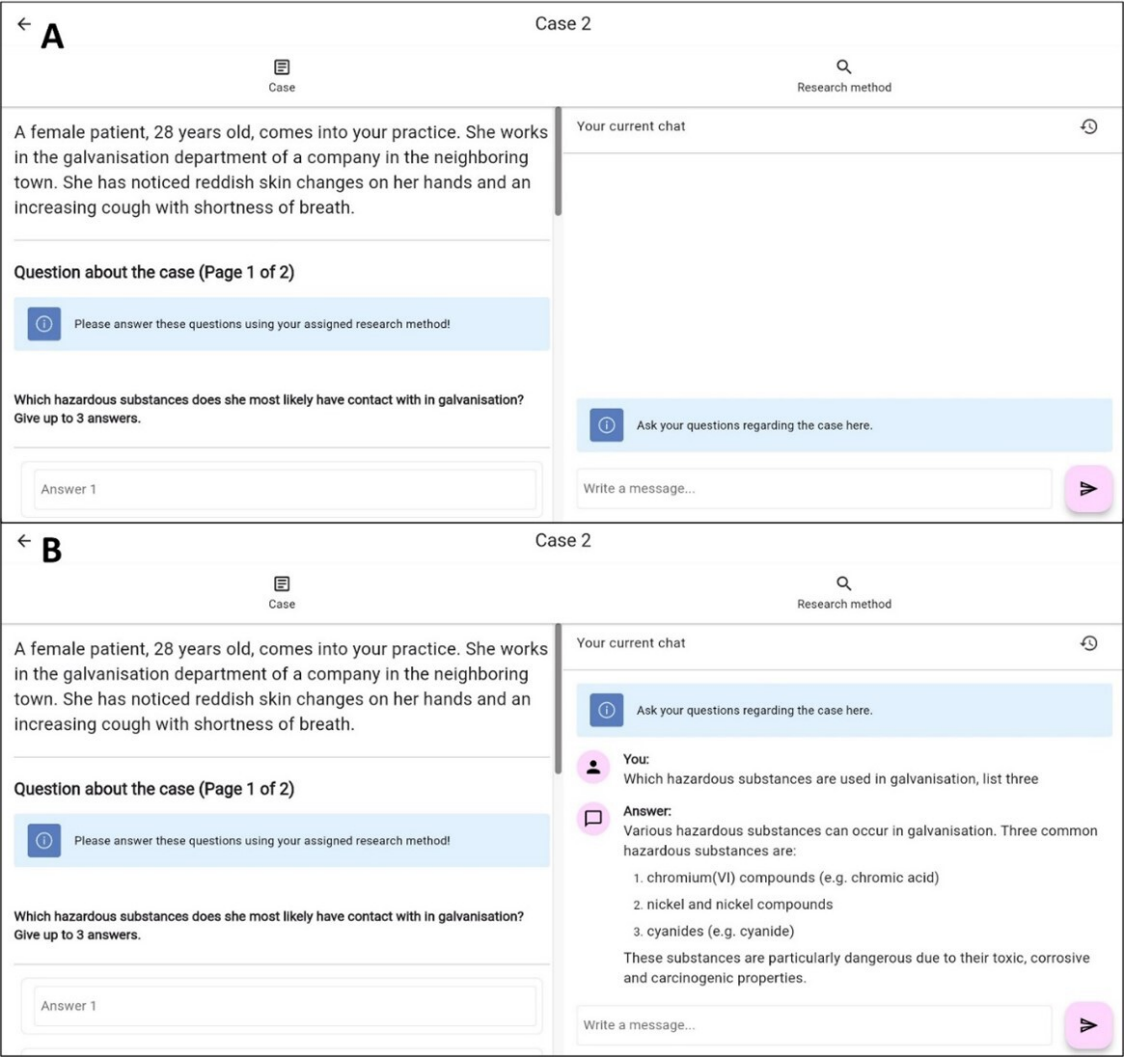
View of the ChatGPT group for solving case 2 before (A) and after (B) input. ChatGPT 4 was connected to the input window..

Exactly 6 questions were asked for each case, which were to be answered with yes or no, multiple choice, or free-text options. For each question, there was also a “don’t know” option. The questions can be viewed in Multimedia Appendix 1. Three questions were always presented on the screen at the same time as the introductory case vignette. Next to it was either a window for integrated ChatGPT input (Figure 2) or the indication that the usual means should be used freely. Each question had to be answered or the “don’t know” option had to be checked. After completing the 3 questions, the user moved on to the next 3. It was then no longer possible to go back and change the answers, and rule out learning effects from the later questions. In order to record how exactly the respondents entered their answers, it was also not possible to copy the questions or the case vignette and paste them into the chat window.

The number of correct answers or “don’t know” answers was counted in the evaluation. For questions that were offered for answering without research before the group assignment (before), a comparison of before and after was also carried out.

After each case, it was possible to choose whether another case should be processed or whether the respondent should be forwarded to the final questions. This was to prevent the final questions from not being processed because the respondents did not want to work on the other cases.

In the final questionnaire, respondents were again asked to assess their occupational medicine expertise and were also asked which research tools were ultimately used. In addition to a rating of the experience of the research method, positive and negative comments were recorded.

The primary outcome was the case performance as indicated by a number of (right) answers recorded from case processing. The secondary outcome was a change in (right) answers for certain questions that were asked before without any support and again during case processing with the group-assigned method and self-assessment of occupational medicine expertise recorded before and after case processing.

### Application

A web-based application with an integrated environment for answering questions, subsequent assignments to a group, and a case view was developed for the study. In addition, an integrated chat window was built into the case view for the ChatGPT cohort. This should look as similar as possible to the ChatGPT user interface. A screenshot can be seen in Figure 2. The development of this application allowed for user-friendly data collection of chat entries and responses during unsupervised study participation. All data was stored in a structured query language database. The LLM was integrated via the Chat Completions application programming interface, a developer interface from OpenAI, the developers of GPT-4 and ChatGPT [12]. Communication with OpenAI took place via our own servers and was carried out in such a way that OpenAI did not receive any information such as the IP addresses of the participants aside from the actual input. The participants’ input and, in the case of ongoing chat, the previous input and responses were used as context for the LLM. The model‘s response was already loaded (“streamed”) into the application in sections during generation to enable the response to be displayed earlier. The most up-to-date and powerful model from OpenAI between March 2024 and May 2024 was used: gpt-4‐0125-preview. Each conversation contained a system prompt. This is a command that gives the model context and instructions for the conversation. The system prompt contained, among other things, an explanation that it is an assistant for doctors and students to investigate occupational disease and should be helpful. The entire system prompt read as follows: "You are a helpful assistant who helps with questions regarding occupational diseases in Germany. You communicate with medical students or doctors. You check your information for accuracy. If there are ambiguities, for example, with abbreviations, ask what exactly is meant. Answer specifically and be brief. If there are any uncertainties, explain them.*”*

### ChatGPT Input

The inputs made by the participants and the outputs generated by ChatGPT were examined by 2 individuals separately and then compared. Differences in the assessment were discussed between the 2 raters and resolved by consensus. In addition to the number of words entered and output, the type of communication on the participants’ side was also recorded, for example, was ChatGPT addressed, was the input in complete sentences or as keywords?

### Ethical Considerations

This study was carried out with the approval of the responsible ethics committee (Ethics Committee of the Medical Faculty of RWTH Aachen University, EK 24‐065) in accordance with the Declaration of Helsinki in its current version and with national law. Informed consent was obtained from all participants. Study data were obtained anonymously without any personal information. Participants received no compensation. Three €25 vouchers (approx. 28 US$) were raffled off among all participants. They could voluntarily provide an email address, without this being linked to the other data. This email address was only used to draw and send the vouchers.

### Statistical Analysis

The data were analyzed using GraphPad Prism (version 10.2.3) and SPSS (version 29.0.0.0; IBM Corp).

No sample size analysis was performed beforehand. The recruitment period was set at 3 months. The aim was to recruit as many patients as possible during this time with at least 50 participants being included.

The data are given as number and percentage, number of answers or correct answers, or mean (SD). Group differences between the ChatGPT group and the group with its own research were examined either by Mann-Whitney *U* test or chi-square test for group sizes of at least 5 persons, otherwise by Fisher exact test . All statistical tests were 2-sided with *P*<.05 as the significance level.

## Results

### Demographics of the Participants

**Figure 3.**
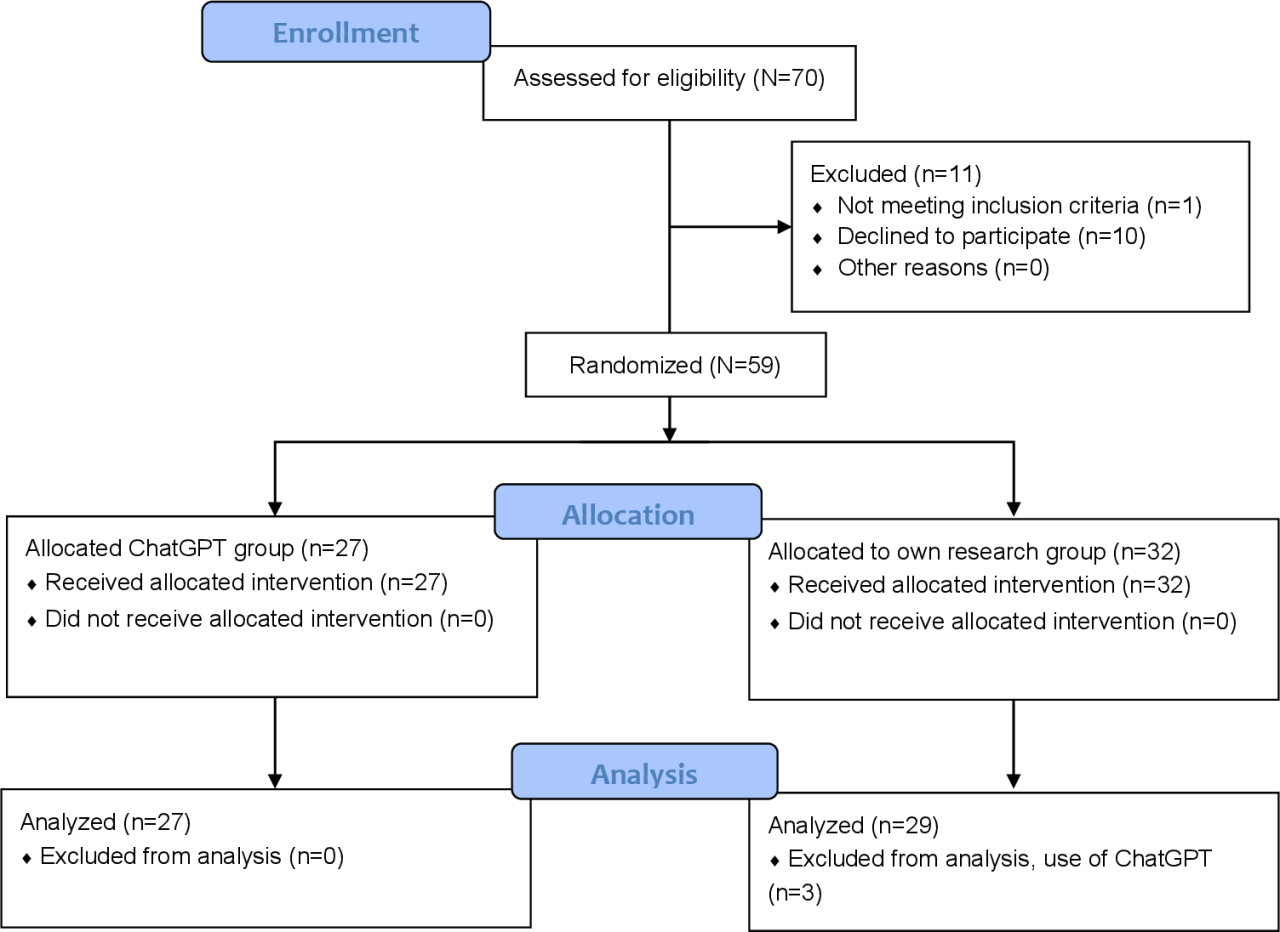
CONSORT (Consolidated Standards of Reporting Trials) flow diagram. After assessment of 70 participants for eligibility, 59 were randomized in 2 groups. 27 participants were allocated to the ChatGPT group of which all were subsequently included in the analysis. 32 participants were allocated to the group with own research. Here, three participants had to be excluded for analysis as they used ChatGPT as research tool.

A total of 70 respondents made entries in the web-based questionnaire (Figure 3). A total of 10 (10/70, 14%) had already dropped out before being assigned to a group. One person (1/70, 1%) did not state whether they had studied medicine or worked as a doctor and 3 people (3/70, 4%) stated after case processing that they had used ChatGPT or similar LLM but had been assigned to the group with research without ChatGPT. They had to be excluded after participation in the study since participants were not informed of this exclusion criterion beforehand to avoid unconscious influence toward the use of ChatGPT. After exclusion, the data of 56 respondents were evaluated (Table 1).

A total of 27 participants, 17 (17/27, 63%) female and 21 (21/27, 78%) students, were assigned to the ChatGPT group. A total of 29 participants, 24 (24/27, 83%) female, and 15 (15/29, 52%) students, used their own research tools. The ChatGPT group had significantly more students than the research group. Overall, the average age was 28.5 (SD 7.2) years and students were on average (SD) in their 9th (1.8) semester. Both groups rated themselves similarly in terms of occupational medicine expertise. The ChatGPT group gave themselves a 3.9 (SD 1) according to school grades (almost a 4, which corresponds to the grade “sufficient”). The research group gave themselves a 3.8 (SD 0.9).

The number of participants was recorded again before each substep; only 28 of 56 participants (28/56, 50%) answered the last case. The same decrease was observed in both groups, meaning that a “loss-to-follow-up” bias is unlikely here due to the research method.

**Table 1.** Demographics of the participants. After completing the sociodemographic and knowledge questionnaire, participants were randomly allocated to the ChatGPT group or to conduct their own research. Data are presented as n (%) or mean value (SD).

	Total	ChatGPT	Own research	*P* value
Sex, n (%)	56	27 (48.2)	29 (51.8)	—^a^
Female	41	17 (63.0)	24 (82.8)	.10^b^
Male	15	10 (37.0)	5 (17.2)	—
Status, n (%)
Students	36	21 (77.8)	15 (51.7)	.046^b^^c^
Physicians	20	6 (22.2)	14 (48.3)	—
In training	12	3 (50.0)	9 (64.3)	—
Specialists	7	2 (33.3)	5 (35.7)	—
Attendings	1	1 (16.7)	0 (0)	—
Occupational medicine	5	2 (33.3)	3 (21.4)	—
Participation, n (%)
Case 1	41	22 (81.5)	19 (65.5)	—
Case 2	32	18 (66.7)	14 (48.3)	—
Case 3	28	15 (55.6)	13 (44.8)	—
Concluding questions	29	16 (59.3)	13 (44.8)	—
Age (years), mean (SD)	28.5 (7.2)	27.3 (7.3)	29.6 (7)	.07^d^
Semester (students), mean (SD)	9 (1.8)	8.9 (1.7)	9.2 (1.9)	.51^d^
Self-assessment (school grades), mean (SD)	3.8 (0.9)	3.9 (1)	3.8 (0.9)	.74^d^
Research tools commonly used, n (%)				
Amboss [10]	45	23 (51.1)	22 (48.9)	—
Doccheck [11]	48	24 (50.0)	24 (50.0)	—
Google	44	20 (45.5)	24 (54.5)	—
UptoDate [13]	8	4 (50.0)	4 (50.0)	—
Wikipedia	26	11 (42.3)	15 (57.7)	—
Thieme eRef [14]	12	5 (41.7)	7 (58.3)	—
Via Medici [15]	3	1 (33.3)	2 (66.7)	—

a —: not applicable.

b Chi-square test.

c *P*<.05. For the number students

d Mann-Whitney *U* test for unpaired samples.

### Case 1: Asbestos-Associated Changes in the Pleura

In terms of the number of correct answers, there was a significantly higher number of correct answers to the question about hazardous substances that can cause pleural changes in the ChatGPT group than after their own research (Tables2  3) which corresponded to a small effect (Cohen *r*<0.5). Only the ChatGPT group was able to significantly increase the number of correct answers to the question about 3 types of cancers caused by asbestos compared to before the group assignment.

With regard to the question of whether a cholangiocellular carcinoma can be recognized as an occupational disease in Germany, there were no group differences, neither between ChatGPT and own research nor between before and after group assignment. Only half as many participants (7/21, 33%) ) were able to make the (correct) decision that an occupational disease should be reported in this case with the support of ChatGPT than with their own research (14/21, 67%). Application of ChatGPT was associated with a 6 times higher probability of indicating to report the occupational disease than with own research.

**Table 2.** Case 1: Gardener with asbestos-associated pleural lesions—free-text questions. Participants were asked 3 questions, listing 3 answers in free text (number of responses). Free-text questions included the instruction to list hazardous substances that can cause pleural changes, materials that can induce them, and types of cancers caused by asbestos exposure. Either ChatGPT or the research method of their own choosing was used, depending on group allocation for research.

Case 1 (n=41)	Total (mean, SD)	ChatGPT (mean, SD)	Own research (mean, SD)	*P* value^a^	Cohen *r*
Hazardous substances with pleural changes, mean (SD)	2.2 (0.8)	2.5 (0.8)	1.8 (0.8)	.01^b^	−0.38
Materials pleural changes, mean (SD)	1.5 (1.2)	1.7 (1.3)	1.3 (1.1)	.35	−0.28
Types of cancers asbestos, mean (SD)	2.8 (0.5)	2.9 (0.5)	2.7 (0.6)	.37	−0.25
Types of cancers before, mean (SD)	2 (0.8)	1.8 (0.8)	2.2 (0.8)	.18	−0.22
Types of cancers (comparison before vs after, *P* value)^c^	<.001^d^	<.001^d^	.06	—^e^	—

a Mann-Whitney *U* test for unpaired samples.

b *P*<.05.

c Wilcoxon test for paired samples.

d *P*<.001.

e —: not applicable.

**Table 3.** Case 1: Gardener with asbestos-associated pleural lesions—multiple-choice questions. Participants were asked 3 questions choosing the right answers from multiple choice. Multiple-choice questions asked whether a CCC^a^ could be officially recognized as an OD^b^ in Germany, which OD number corresponds to asbestos-induced pleural changes, and whether an official OD report should be filed. Either ChatGPT or the research method of their own choosing was used, depending on group allocation for research.

Case 1 (n=41)	Total	ChatGPT	Own research	*P* value^c^	OR^d^ (95% CI)
CCC as OD	30	16	14	.95	1.05 (0.26‐4.20)
CCC as OD before	19	10	9	.90	1.08 (0.32‐3.70)
CCC as OD (comparison before versus after as difference)	11	6	5	—^e^	—
CCC as OD “Don’t know”	3	1	2	—	2.47 (.21‐29.63)
CCC as OD “Don’t know” before	18	9	9	.68	1.30 (0.38‐4.49)
CCC as OD “Don’t know” (comparison before versus after as difference)	–15	–8	–7	—	—
Multiple-choice OD (pleural changes asbestos)	36	20	16	.51	0.53 (0.08‐3.59)
OD report	21	7	14	.007^f^	6.00 (1.54‐23.36)

a CCC: cholangiocellular carcinoma.

b OD: occupational disease.

c Chi-square test or Fisher exact test if fewer than 5 per group.

d OR: odds ratio.

e —: not applicable.

f *P*<.01.

### Case 2: Galvanization-Associated Occupational Allergy

There were no relevant differences between groups for the correct listing of hazardous substances in galvanization, only a general learning effect in contrast to the time before group assignment (Tables4  5). Participants in the ChatGPT group named the correct next diagnostic steps significantly more often (medium effect; Cohen *r*<0.8). They were able to list more correct fields of activity in which occupational asthma can develop (medium effect; Cohen *r*<0.8), although they were able to state fewer from memory than the other group in the query before group assignment (medium effect; Cohen *r*<0.8).

There were no group differences in the questions about the suspected diagnosis, the correct occupational disease according to the German ordinance on occupational diseases, and whether a report should be made, although the proportion of correct answers was rather low in both groups.

**Table 4.** Case 2: Allergy in galvanization—free-text questions. Participants were asked 3 questions, listing 3 answers in free text (number of responses) or choosing the right answers from multiple choice. Free-text questions included the instruction to list hazardous substances workers could be exposed to in galvanization, the next steps in the diagnostic procedure for the patient in case 2, and in which occupational fields occupational asthma could occur. Either ChatGPT or the research method of their own choosing was used, depending on group allocation for research.

Case 2 (n=32)	Total (mean, SD)	ChatGPT (mean, SD)	Own research (mean, SD)	*P* value^a^	Cohen *r*
Hazardous substances galvanization	2.9 (0.4)	3 (0)	2.8 (0.6)	.18	–0.41
Hazardous substances galvanization before	0.7 (1.1)	0.5 (1)	1 (1.2)	.18	—^b^
Hazardous substances galvanization (comparison before versus after; p^§^)	<.001^c^	<.001^c^	<.001^c^	—	—
Diagnostic procedure	2.2 (0.6)	2.5 (0.5)	1.9 (0.6)	.02^d^	–0.62
Occupational fields allergic asthma	2.9 (0.4)	3 (0)	2.7 (0.5)	.03^d^	–0.56
Occupational fields allergic asthma before	2.1 (0.9)	1.8 (1.1)	2.5 (0.5)	.049^d^	–0.54
Occupational fields allergic asthma (comparison before versus after; *P* value)^e^	<.001^c^	.002	.25	—	—

a Mann-Whitney *U* test for unpaired samples.

b —: not applicable.

c *P*<.001.

d *P*<.05.

e Wilcoxon test for paired samples.

**Table 5.** Case 2: Allergy in galvanization—multiple-choice questions. Participants were asked 3 questions choosing the right answers from multiple choice. Multiple-choice questions asked for the suspected diagnosis, the corresponding occupational disease, and whether an official occupational disease report should be filed. Either ChatGPT or the research method of their own choosing was used, depending on group allocation for research.

Case 2 (n=32)	Total, n	ChatGPT, n	Own research, n	*P* value^a^	OR^b^ (95% CI)
Suspected diagnosis	15	9	6	.69	0.75 (0.18‐3.06)
Occupational disease	14	8	6	.93	0.94 (0.23‐3.84)
Occupational disease report	28	15	13	.42	2.6 (0.24‐28.15)

a Chi-square test for the number of correct answers..

b OR: odds ratio.

### Case 3: Dental Technician With Berylliosis

In both groups, approximately the same number of hazardous substances could be listed to which the patient was exposed as a dental technician (Tables6  7). There were also no group differences between the ChatGPT and own research or before and after group assignment with regard to occupational fields for potentially causing berylliosis. Both groups learned more by using their research tools.

The participants in both groups also answered the question of whether sarcoidosis can be recognized as an occupational disease equally often correctly, even when comparing before and after group assignment. Participants in the group with their own research were able to correctly identify the case as occupational disease no. 1110 according to the German ordinance on occupational diseases (“diseases caused by beryllium or its compounds”) more often than users of ChatGPT. Given the asymmetrical distribution of the groups among the responses, no realistic estimate of the odds ratio could be obtained. The lymphocyte transformation test and the need to report this occupational disease were reported by the same number of participants in both groups, albeit few.

**Table 6.** Case 3: Dental technician with berylliosis—free-text questions. Participants were asked 2 questions listing 3 answers in free text (number of responses. Free-text questions included the instruction to list hazardous substances dental technicians could be exposed to and occupational fields where occupational asthma could occur. Either ChatGPT or the research method of their own choosing was used, depending on group allocation for research.

Case 3 (n=28)	Total (mean, SD)	ChatGPT (mean, SD)	Own research (mean, SD)	*P* value^a^	Cohen *r*
Hazardous substances: Dental technician	2.8 (0.5)	2.9 (0.5)	2.8 (0.4)	.31	–0.51
Occupational fields berylliosis	1.6 (0.7)	1.4 (0.8)	1.8 (0.6)	.21	–0.55
Occupational fields berylliosis before	0.3 (0.9)	0.2 (0.8)	0.5 (1)	.31	–0.52
Occupational fields berylliosis (comparison before versus after; *P* value)^b^	<.001^c^	<.001^c^	<.001^c^	—^d^	—

a Mann-Whitney *U* test for unpaired samples.

b Wilcoxon test for paired samples.

c *P*<.001.

d —: not applicable.

**Table 7.** Case 3: Dental technician with berylliosis—multiple-choice questions. Participants were asked 4 questions choosing the right answers from multiple choice. Multiple-choice questions asked whether sarcoidosis could be officially recognized as an OD^a^ in Germany, which OD number berylliosis corresponds to in Germany, which diagnostic tests could be used, and whether an official occupational disease report should be filed. Either ChatGPT or the research method of their own choosing was used, depending on group allocation for research

Case 3 (n=28)	Total, n	ChatGPT, n	Own research, n	*P* value^b^	OR^c^ (95% CI)
Sarcoidosis as OD	23	13	10	.50	0.51 (0.07‐3.68)
Sarcoidosis as OD before	11	4	7	.14	3.2 (0.66‐15.59)
Sarcoidosis as OD (comparison before versus after as difference)	12	9	3	—^d^	—
Sarcoidosis as OD “Don’t know”	0	0	0	—	—
Sarcoidosis as OD “Don’t know” before	5	4	1	.19	0.23 (0.02‐2.38)
Sarcoidosis as OD “Don’t know” (comparison before versus after as difference)	–5	–4	–1	—	—
OD berylliosis	8	1	7	.006^e^	16.33 (1.63‐163.44)
Diagnostic test	13	5	8	.14	3.20 (0.68‐15.07)
OD report	20	9	11	.15	3.21 (0.66‐15.59)

a OD: occupational disease.

b Chi-square test or Fisher's exact test if fewer than 5 per group.

c OR: odds ratio.

d —: not applicable.

e *P*<.01.

### Concluding Questions

Even though there was a tendency toward higher satisfaction in the ChatGPT group, there were no significant differences between the 2 groups (Table 8). The ChatGPT group showed a significantly greater increase in self-assessment than the group with their own research.

The participants in the group with their own research primarily used DocCheck and Google, with a few also using Amboss and Wikipedia. Participants who indicated ChatGPT here were completely excluded from the analysis.

**Table 8.** Concluding questions—survey on satisfaction and self-assessment before and after the cases. Participants were asked about their satisfaction with the research method, as well as their self-assessment after case processing. For participants who provided a self-assessment in the final questionnaire, their corresponding self-assessment before case processing was compared. Either ChatGPT or the research method of their own choosing was used, depending on group allocation for research. Participants in the own research group were asked which tools they used.

Concluding questions (n=29)	Total	ChatGPT	Own research	*P* value^a^	Cohen *r*
Satisfaction with research method, mean (SD)	3.1 (1.2)	2.8 (1.3)	3.4 (0.9)	.14	−0.57
Self-assessment OME^b^ before, mean (SD)	3.7 (.8)	3.9 (.9)	3.5 (0.8)	.12	−0.56
Self-assessment OME after, mean (SD)	3.4 (1)	3.4 (1.1)	3.4 (0.9)	.92	−0.44
Self-assessment OME (comparison before vs after), *P* value^c^	.10	.047^e^	.99	—^d^	—
Used research tools, n (%)					
Amboss	5	0 (0)	5 (100)	—	—
DocCheck	10	0 (0)	10 (100)	—	—
Google	13	0 (0)	13 (100)	—	—
Wikipedia	2	0 (0)	2 (100)	—	—

a Mann-Whitney *U* test for unpaired samples.

b OME: occupational medicine expertise.

c Wilcoxon test for paired samples.

d —: not applicable.

e *P*<.05

### Evaluations of the Participants

After using the tool, participants were able to voluntarily leave positive and negative feedback. In the

ChatGPT group, the simple and practical use, as well as the possibility of follow-up questions, was noted positively several times. It was also praised that the answer was displayed in detail with reasons so that something additional was learned and there was the opportunity to ask very specific questions. On the negative side, the time it took to provide a complete answer was mentioned several times, as was the lack of references.

There were no positive comments in the group on their own research; negative comments were that the internet search was laborious and time-consuming.

In general, many participants requested a solution for each question with the right answer. This was not given so that there could be no “contamination” of the study population, for example, within a semester.

### Analysis of ChatGPT Input

In the ChatGPT group, there were 2 participants who only entered keywords, similar to a Google search. The rest corresponded with ChatGPT like a person and gave explicit instructions in full sentences.

The average number of entered instructions to ChatGPT increased from 5.8 (2.7 SD) messages in case 1 to 6.3 (SD 2.3) to 10.5 (SD 4.2) messages.

## Discussion

This study investigated how a generative LLM such as ChatGPT can support medical research and clinical decision-making and how it performs in comparison to conventional research. The subject of occupational lung diseases was selected in accordance with the stipulations of German occupational disease legislation. Approximately 80% of deaths from occupational diseases in Germany are caused by lung diseases [16]. In the processing of the cases, it was demonstrated that ChatGPT facilitated the participants‘ ability to conduct targeted research, such as identifying potential hazardous substances or activities, and enhanced their self-assessment of their specialist knowledge. However, clinical decisions, such as determining whether an occupational disease report should be filed, were more frequently made correctly through the participants’ independent research.

This project was initiated in response to the recognition that numerous medical professionals encounter difficulties in navigating the complexities of occupational disease, including uncertainty about its existence and the appropriate reporting procedures. During the course of everyday clinical practice, there was a clear indication of a need for a dedicated digital resource to address these challenges. In Germany, there is a web-based search tool provided by the German Social Accident Insurance [17]. One can enter the diagnosed disease according to the ICD-10 code and receive possible occupational diseases. However, it is solely based on the presenting disease and has no possibility to put in certain chemicals or exposures. From experience in our university hospital, the majority of medical students and physicians in other fields than occupational medicine do not know it. With the introduction of ChatGPT 4, an enhanced LLM with a more substantial data foundation and augmented performance, coupled with its internet connectivity via Bing, which should markedly curtail the proclivity for confabulation, has made a corresponding function via ChatGPT a realistic prospect for the first time [18,19]. The exclusion of 3 participants due to the use of ChatGPT illustrates the growing prevalence of ChatGPT as a search engine and database. This phenomenon appears to be particularly pronounced among younger individuals [20]. The 3 individuals excluded from our study were all students under the age of 30.

Similar to the results of this study, other studies that used ChatGPT to answer medical questions showed that ChatGPT can answer many correctly. In 1 study, ChatGPT 4 was even able to answer more ophthalmology questions correctly than ChatGPT 3.5 and a human comparison group [21]. ChatGPT answered questions about diagnoses and differential diagnoses for medical case vignettes with an acceptable but not yet good level of accuracy [22].

In regard to clinical decision-making, ChatGPT has been demonstrated to be less effective than medical experts in the present context. In this study, the group that used their customary research techniques was significantly more successful in making accurate clinical decisions than the ChatGPT group. It seems probable that the discrepancy can be attributed to implicit considerations on the part of the participants, given that ChatGPT provides a definitive ”yes“ or ”no“ response to the posed question, whereas the participants in their own research are required to arrive at a decision independently. Probably, considerations such as the fact that imaging with ionizing radiation should be avoided in a young woman in case 2 were not given sufficient weight by ChatGPT. A comparable outcome was observed in the study conducted by Zaboli et al [23], which compared the triage decisions made by specialized triage nurses in the emergency department with those generated by ChatGPT. Here, the triage nurses demonstrated a significantly superior performance compared to ChatGPT. In a separate study, ChatGPT was tasked with answering questions from specialist orthopedic examinations in the United States. The applicable knowledge demonstrated by ChatGPT 4 was found to be comparable to that of an individual in their third year of orthopedic training (residency) [24]. Furthermore, ChatGPT has demonstrated the capacity to respond to queries and offer diagnoses in urological matters at the level of medical practitioners in training, while human expertise is more proficient in more complex scenarios [25]. Interestingly, ChatGPT 4 also exhibits inferior performance compared to human experts in radiological disciplines, despite the frequent assertion that it is particularly well-suited for use in this domain. For instance, the proficiency of radiology professionals in advanced training was not reached in severe neuroradiological cases [26]. In the United States Medical Licensing Examination, ChatGPT performed comparably to a third-year medical student [27,28].

In terms of standardized tasks, however, ChatGPT appears to be highly functional. In the evaluation of standard ECGs, ChatGPT outperformed both emergency physicians and cardiologists. For more challenging questions, it demonstrated a level of proficiency comparable to that of cardiologists and surpassed the performance of emergency physicians [29]. Similarly, the generation of information letters for patients yielded comparable outcomes. In this context, the letters created by ChatGPT were rated more highly by patients and physicians than those designed by surgeons [30]. Furthermore, ChatGPT 4 is capable of providing satisfactory responses to radiology-related patient queries [31,32].

The quality of the LLM’s output is contingent upon the quality of the training data. It seems reasonable that in the context of occupational medicine, and particularly in the specific case of German occupational disease law, the training data available for the purpose of training the models was likely limited. Specific training with information relevant to occupational medicine could enhance the application but was not available at the time. Information on German occupational diseases are typically accessible on the web, though predominantly in German. Specially developed LLM for the medical context, such as Med-Palm from Google [33,34] represent potential improvements but were not considered in this study due to a lack of accessibility.

In this study, ChatGPT demonstrated significant difficulties in finding a solution to case 3, a dental technician with berylliosis. This case was selected for this study due to its rarity in Germany, where it is not frequently recognized or reported [9]. During case processing, confabulations and false statements were recorded in the output. In a multiple-choice question in which the potential occupational disease was to be selected from five different ones with the initial digit 11 (prefix for occupational diseases caused by metals and metalloids), ChatGPT regularly stated that none of the offered options were correct. Furthermore, it proposed occupational diseases numbered 4103 (asbestos dust lung disease, also known as asbestosis) or 4104 (lung cancer, laryngeal cancer, or ovarian cancer caused by asbestos dust). Furthermore, ChatGPT 4 even invented sources that did not exist.

As with any innovation, its use in a medical context must be subjected to rigorous scrutiny and it is to be expected that errors in the LLM will occur. The responses to the use of ChatGPT were noteworthy. The participants exhibited an enhancement in their self-assessment of their own specialist knowledge. Nevertheless, there was a tendency for participants in the group who conducted their own research to report a higher level of self-assessment. In conclusion, both groups awarded themselves an identical rating following the case processing, with an average grade of 3.4 (satisfactory). Additionally, there was a tendency toward greater satisfaction with the use of ChatGPT. This is evidenced by the positive ratings given to the ease and practicality of its use, as well as the possibility of posing follow-up queries. Conversely, the negative ratings assigned to the working time of ChatGPT were attributed to the system requirements at the time. However, with ChatGPT 4o, a significantly faster system with comparable quality is now available.

This study evaluated the performance in only 3 cases with as little as 6 questions per case. It was designed as a pilot study to assess whether ChatGPT can be used for occupational medicine cases. As a further limitation, only occupational disease cases related to the lungs were included. Whether the usability could be extended for the entire field of occupational diseases cannot be assessed in this study and should be addressed in future studies.

In addition to the number of cases, the lack of monitoring of the group with its own research represents a further limiting factor in this study. Neither the entries nor the sources were subjected to any form of verification. For example, it is possible that a greater number of individuals may have used ChatGPT without disclosing this information. This study was designed with the explicit intention of testing against the conventional research methodology. Consequently, the typical applications and devices were also employed to ensure that the approach was as realistic as possible. With regard to the relatively small number of participants, recruitment proved challenging, and only approximately half of the participants engaged with the study until the conclusion of the final case. The lengthy processing time was identified as the primary reason for withdrawal. Unfortunately, processing time was not recorded. Thus, it cannot be assessed in detail if and how the processing time affected dropout. In some cases, the participants expressed a desire to conduct further research until all questions had been answered correctly. This could also have resulted in a positive distortion of performance. The number of correct answers could have been lower had the participants been prompted to provide answers after their initial brief research. As both groups were equally decimated over the cases, a “lost-to-follow-up bias” seems unlikely due to the research method. The two groups were formed by randomization using a digital weighted coin. In the case of an unequal proportion of students and doctors, the coin was weighted in such a way that it was then more likely to be assigned to the other group, but never simply assigned. Nevertheless, there was a certain unequal distribution of doctors across the groups, so they were more represented in the group with their own research. Physicians can be assumed to have greater expertise and experience, but the proportion of colleagues working in occupational medicine was evenly distributed. Nevertheless, a certain bias may have taken place here, so that regardless of the research method, the performance of the group with more physicians was greater, for example, with regard to clinical decisions. In future surveys, this should be taken into account even more and a higher number of participants should generally be included. Nevertheless, this study is one of the few that examines the realistic use of ChatGPT with the input of end users and does not only assess data or questions as input in ChatGPT. The use of ChatGPT by precisely these users, namely medical students and doctors, is conceivable and is already happening to some extent today in the clinical routine. Although no significant differences between the 2 groups could be observed, female participants made up the majority of participants in the study overall. There is a sex disparity in the use of generative LLM tools usually favoring men. A survey conducted by the Bank for International Settlements stated that 50% of men reported using generative artificial intelligence over the previous 12 months, compared to only 37% of women [35]. However, women engage with chatbots in a more relational and exploratory manner than men, for example, asking follow-up questions and seeking clarification [36]. A recent large meta-analysis even reported a female lead in digital knowledge and skills among students [37]. However, the manner in which e-learning and LLM tools are used is shaped not only by sex but also by age, background, and prior experience [38-40]. The effects of those factors could not be investigated in this setup but should be addressed and examined in future studies.

In conclusion, it can be stated that ChatGPT 4 is a valuable tool for targeted medical research, even for highly specific questions in occupational medicine concerning occupational diseases in Germany. However, it is imperative that clinical decisions are not based on the output of the LLM, but also assessed by qualified medical professionals. The potential of the LLM to perform well after training with relevant data or overviews regarding occupational diseases and specialized occupational medicine instructions (prompts) remains uncertain and requires further investigation.

## Supplementary material

10.2196/63857 Multimedia Appendix 1 Socio-demographic questionnaire.

10.2196/63857 Checklist 1 CONSORT-eHEALTH checklist (V 1.6.1).

## Abbreviations

LLM: large language model
